# Analysis of the Transcriptome Provides Insights into the Photosynthate of Maize Response to Salt Stress by 5-Aminolevulinic Acid

**DOI:** 10.3390/ijms26020786

**Published:** 2025-01-17

**Authors:** Ying Jiang, Min Li, Yumei Qian, Hao Rong, Tao Xie, Shanshan Wang, Hong Zhao, Liangli Yang, Qingyun Wang, Yanyong Cao

**Affiliations:** 1 School of Biological and Food Engineering, Suzhou University, Suzhou 234000, China; bazhujiangying@126.com (Y.J.); liminzyl@sina.com (M.L.); qianyumei@ahszu.edu.cn (Y.Q.); ronghao@ahsuz.edu.cn (H.R.); 007521@yzu.edu.cn (T.X.); 18853818778@163.com (S.W.); swx04239zh@126.com (H.Z.); yliangli2019@163.com (L.Y.); 2 Anhui Province Key Laboratory of Farmland Ecological Conservation and Pollution Prevention, Anhui Agricultural University, Hefei 230036, China; 3 Institute of Cereal Crops, Henan Academy of Agricultural Sciences, Zhengzhou 450002, China

**Keywords:** salt stress, *Zea mays* L., 5-aminolevulinic acid, photosynthesis, transcriptome

## Abstract

Salt stress is a significant environmental factor that impedes maize growth and yield. Exogenous 5-aminolevulinic acid (ALA) has been shown to mitigate the detrimental effects of various environmental stresses on plants. However, its regulatory role in the photosynthesis mechanisms of maize seedlings under salt stress remains poorly understood. Transcriptome sequencing and physiological index measurements were conducted on the leaves of the “Zhengdan 958” cultivar subjected to three different treatments. Differential expression analysis revealed 4634 differentially expressed genes (DEGs), including key transcription factor (TF) families such as NAC, MYB, WRKY, and MYB-related, across two comparisons (SS_vs_CK and ALA_SS_vs_SS). Significant enrichment was observed in the metabolic pathways related to porphyrin metabolism, photosynthesis-antenna proteins, photosynthesis, and carbon fixation in photosynthetic organisms. ALA treatment modulated the expression of photosynthesis-related genes, increased photosynthetic pigment content, and enhanced the activities of superoxide dismutase (SOD) and catalase (CAT), thereby mitigating the excessive accumulation of reactive oxygen species (ROS). Furthermore, ALA increased starch content under salt stress. These findings establish a foundational understanding of the molecular mechanisms through which ALA regulates photosynthesis under salt stress in maize seedlings. Collectively, exogenous ALA enhances maize’s salt tolerance by regulating photosynthesis-related pathways.

## 1. Introduction

Soil salinity, a major abiotic stress, affects both irrigated and non-irrigated agricultural lands and significantly limits global crop productivity. It is predicted that by 2050, up to 50% of arable land will be lost due to soil salinity [1]. Salt stress impairs plant growth by altering rhizosphere osmotic pressure, reducing the availability of water and nutrients, and limiting sustainable agricultural development [2]. Additionally, salt stress induces an increase in reactive oxygen species (ROS) accumulation and elevated concentrations of ions (Na^+^ and Cl^−^), which leads to osmotic stress and ion toxicity [3]. Consequently, osmotic stress triggers a range of physiological and biochemical disruptions, including nutritional imbalances, damage to ROS detoxification mechanisms, alterations in cellular metabolism, membrane damage, and reduced photosynthetic activity [4]. Excessive ion accumulation in chloroplasts can alter organelle ultrastructure, degrade chloroplast pigments, disrupt chloroplast integrity, and impair photosynthetic efficiency, ultimately hindering the photosynthetic process [2,4,5].

Maize (*Zea mays* L.), a globally significant cereal crop, is grown in numerous countries and is widely consumed by both humans and livestock, the loss of global maize production accounts for about 11% of the total production, which is affected by diseases and abiotic stress factors [6]. As a notable C_4_ plant, maize exhibits moderate sensitivity to salinity, which negatively impacts its growth and yield [1,6]. Salt stress induces ion toxicity, significantly reducing seed germination rates, affecting root physiological processes [7], decreasing relative water content (RWC) [8], causing Na^+^ accumulation in tissues, and impeding maize seedling development [9,10]. Salt also induces osmotic stress, which, in turn, affects ABA accumulation, leading to stomatal closure, inhibition of photosynthetic electron transport, generation of ROS, a substantial reduction in photosynthetic activity, and ultimately growth inhibition [11,12]. Furthermore, salt stress reduces the absorption of water by roots and leads to over-production of toxic ions, resulting in excessive ROS accumulation that causes severe oxidative damage [13], disrupts normal cellular homeostasis, induces membrane lipid peroxidation, and inhibits the growth and development of the plant [11,14]. In response, plants synthesize osmoregulatory compounds and antioxidants to mitigate osmotic and oxidative stress, thereby enhancing salt tolerance [13,14]. Moreover, salt stress regulates the expression of various genes, including protein kinases and transcription factors, to improve plant tolerance to stress [15,16]. Currently, a promising strategy to alleviate the adverse effects of salt stress involves the application of plant growth regulators (PGRs) to modulate plant physiological and molecular defense mechanisms.

5-aminolevulinic acid (ALA), a promising PGR, is a key precursor in the biosynthesis of tetrapyrrole compounds such as heme, chlorophyll (Chl), and vitamin B12. These compounds are critical for plant photosynthesis and cellular energy metabolism [17]. ALA holds significant potential for agricultural applications, playing a pivotal role in regulating the expression of photosynthesis-related genes, enhancing photosynthetic efficiency, mitigating ROS accumulation, modulating the antioxidant enzyme system and osmolyte levels, adjusting nitrogen metabolism, altering ion distribution, improving root morphology, and accelerating plant growth. It has been reported to enhance plant tolerance to various abiotic stresses, including chilling stress in pepper [18], drought stress in wheat [19], heat stress in cucumber [20], salt stress in common buckwheat [21] and watermelon [22], pesticide stress in bean [23], low light in watermelon [24], and heavy metal stress in oilseed rape [25]. ALA is believed to actively contribute to antioxidant defense mechanisms, photosynthesis, and other stress-related processes in plants exposed to salt stress, playing an essential role in enhancing salt tolerance [17].

Currently, no comprehensive study exists on the regulation of photosynthetic mechanisms in maize seedlings under salt stress by ALA. This study aims to investigate the effects of ALA on photosynthetic metabolic pathways, the expression of photosynthesis-related genes, and key photosynthetic physiological parameters under salt stress. By focusing on photosynthesis, this work offers new insights into how ALA can enhance maize tolerance to salt stress through the regulation of differentially expressed genes (DEGs) and physiological metabolic processes related to photosynthesis.

## 2. Results

### 2.1. Phenotypic Changes and Microstructure Observation of Maize Response to Salt Stress by ALA

**Figure 1:**
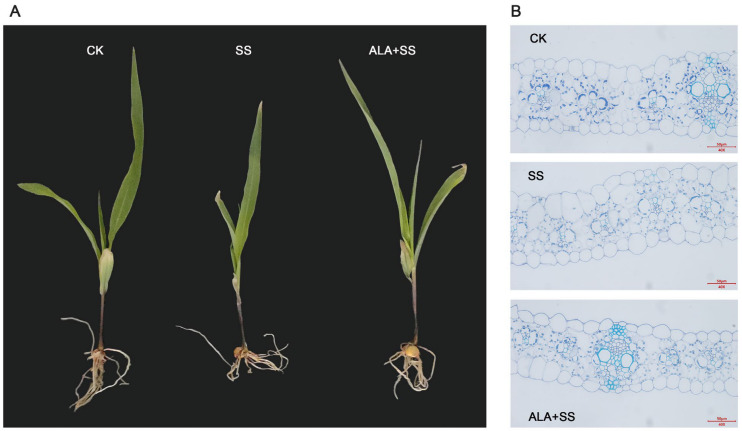
Effect of ALA on (**A**) phenotype and (**B**) microstructure of maize leaves under salt stress.

The effect of salt stress on the phenotypic characteristics of maize leaves was significant. As shown in Figure 1A, after 72 h of exposure to 300 mmol/L NaCl, maize seedlings exhibited stunted growth, smaller leaves, and severe wilting of the first leaf. Exogenous supplementation with ALA alleviated the detrimental effects of salt stress, promoting better growth in the plants.

Through paraffin section, it can be observed that the leaf microstructure of maize seedlings treated with CK exhibited uniform thickness, with an orderly arrangement of mesophyll cells (MCs), clearly visible bundle sheath cells (BSCs), large and well-defined wreath structures, and many chloroplasts in BSCs that were tightly attached to the outer cell membrane of the BSC. In contrast, under salt stress, the leaf microstructure was disrupted, the wreath structure was not obvious, and the BSCs were loosely stacked with fewer and irregularly arranged chloroplasts. Compared with salt stress alone, in maize leaves treated with ALA under salt stress, however, the cells were tightly arranged, the wreath structure and BSCs were clearly defined, the leaf structure was uniform, and the number of chloroplasts in BSCs increased and arranged in a centrifugal manner (Figure 1B).

### 2.2. Transcriptome Data Analysis and DEG Screening of Maize Response to Salt Stress by ALA

To investigate the gene expression profiles of maize seedlings under salt stress regulated by ALA, transcriptome sequencing was conducted on leaf samples from three treatment groups (CK, SS, and ALA_SS), each with three biological replicates. The sequencing yielded a total of 419,955,476 raw reads across the nine samples, with 413,920,888 high-quality clean reads after filtering out joint sequences, low-quality sequences, and reads containing N. The average valid ratio for each sample was 98.56%. Mapping to the maize reference genome Zm-B73-REFERENCE-NAM-5.0 showed an average alignment rate of 88.49%, and the GC content of the library exceeded 52.24%. Sequencing quality control metrics, Q20 and Q30 values were higher than 97.26% and 94.98%, respectively, which confirmed that the data met the standards for subsequent bioinformatics analysis (Table S1).

**Figure 2:**
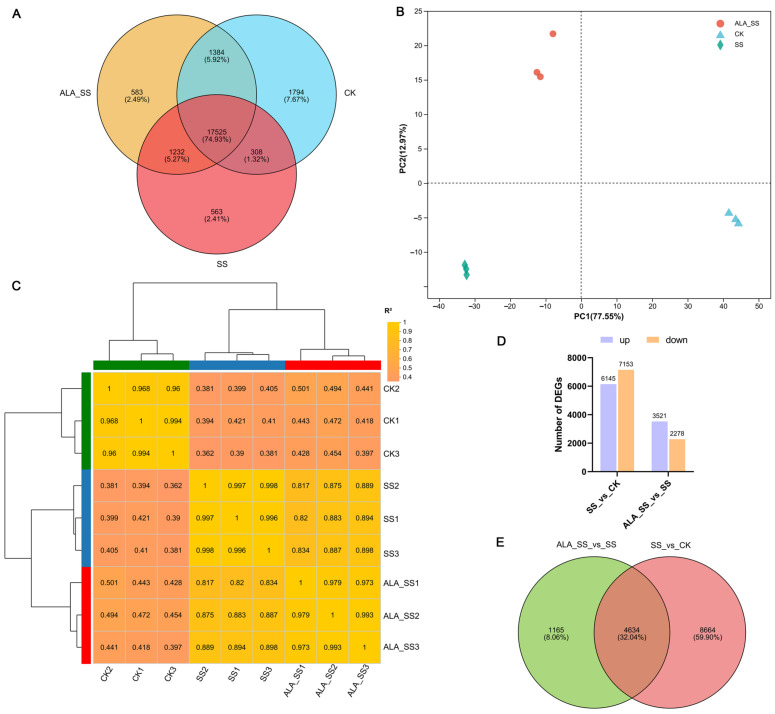
Analysis of transcriptome sequencing data. (**A**) Venn plot of the number of expressed genes in three sample groups. (**B**) Principal component analysis (PCA) based on gene expression profiles of three sample groups. (**C**) Heatmap of the gene expression correlation for all samples. Green box represents the CK treatment group. Blue box represents the SS treatment group. Red box represents the ALA_SS treatment group. (**D**) Histograms of the number of up- and down-regulated DEGs in two comparisons (SS_vs_CK and ALA_SS_vs_SS). (**E**) The Venn diagram of DEGs in two comparisons.

The CK, SS, and ALA_SS treatment groups identified 21,011, 19,628, and 20,724 genes, respectively, with 17,525 genes identified across all three groups, comprising 74.93% of the total (Figure 2A). Principal component analysis (PCA) revealed distinct separation between groups, with good repeatability across biological replicates (Figure 2B). Notably, CK was separated from the other groups along PC1 (77.55%), and ALA_SS was separated from the others along PC2 (12.97%). Correlation analysis of the samples and biological replicates corroborated the PCA results, further validating the experimental design (Figure 2C).

DEGs were identified in two pairwise comparisons: SS_vs_CK and ALA_SS_vs_SS (Figure 2D). In the SS_vs_CK comparison, 13,298 DEGs were identified, with 6145 up-regulated and 7153 down-regulated. In the ALA_SS_vs_SS comparison, 5799 DEGs were identified, with 3521 up-regulated and 2278 down-regulated. A volcano plot was used to visualize the data and highlight the DEGs (Figure S1). Additionally, shared and unique genes were identified between the two comparisons, with 4634 shared genes (32.04%) and 8664 and 1165 unique genes (59.90% and 8.06%, respectively) (Figure 2E). Details of DEG levels in the SS_vs_CK comparison and ALA_SS_vs_SS comparison are shown in Table S2 and Table S3, respectively. Therefore, genes that were co-regulated across both comparisons were examined to elucidate the role of ALA in regulating salt stress in maize.

### 2.3. Functional Annotation and Enrichment Analysis of DEGs

Functional annotation and enrichment analysis of the DEGs were performed using the GO and KEGG databases to explore the roles of shared DEGs between the two comparison groups.

**Figure 3:**
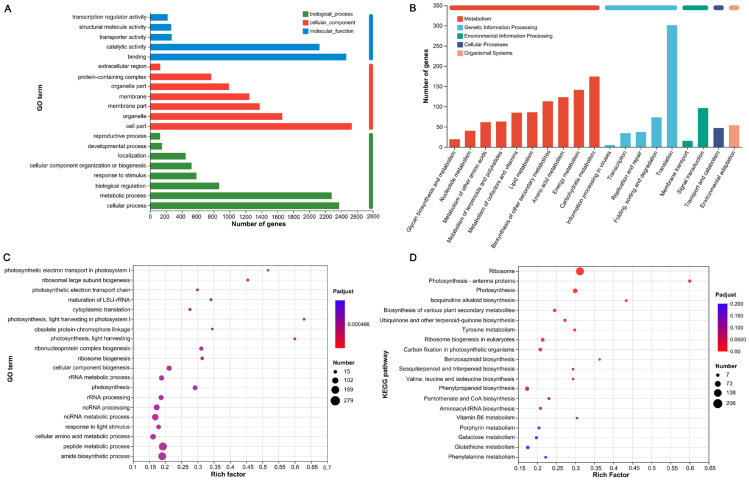
Functional annotation and enrichment analysis of shared DEGs conducted by GO and KEGG. (**A**) A histogram of the GO annotation analysis. (**B**) A histogram of the KEGG annotation analysis. (**C**) A bubble diagram of the GO enrichment analysis. (**D**) A bubble diagram of the KEGG enrichment analysis.

As shown in Table S4, the genes were categorized into biological process (BP), cellular component (CC), and molecular function (MF) terms within the GO framework (Figure 3A). In the BP classification, “cellular processes” (GO: 0009987) encompassed the largest number of DEGs, with 2375 genes, followed by “metabolic processes” (GO: 0008152) with 2280 DEGs. A total of 867 DEGs were annotated under “biological regulation” (GO: 0065007). In the CC classification, the terms “cell part” (GO: 0044464), “organelle” (GO: 0043226), and “membrane part” (GO: 0044425) contained 2533, 1661, and 1377 DEGs, respectively. In the MF category, 2465 DEGs were enriched in “binding” (GO: 0005488), and 2127 DEGs were associated with “catalytic activity” (GO: 0003824). These results suggest that ALA primarily regulated DEGs related to cellular functions, metabolic processes, and biological regulation under salt stress.

KEGG annotation analysis classified the functions of genes into five major categories: metabolism, genetic information processing, environmental information processing, cellular processes, and organismal systems (Figure 3B). Among these, the metabolism category contained the highest number of annotated pathways (100), followed by 22 pathways related to genetic information processing (Table S5). Notably, 174 DEGs were enriched in 15 metabolic pathways within carbohydrate metabolism, including starch and sucrose metabolism (map00500), amino sugar and nucleotide sugar metabolism (map00520), and glycolysis/gluconeogenesis (map00010). Additionally, 141 DEGs were enriched in six energy metabolism pathways, such as photosynthesis (map00195), carbon fixation in photosynthetic organisms (map00710), and photosynthesis-antenna proteins (map00196), indicating that ALA played a role in regulating photosynthesis in maize under salt stress.

To further understand the key biological processes, the top 20 metabolic pathways enriched in the shared DEGs from both comparisons were analyzed through GO and KEGG enrichment, with the lowest P-adjustment values. In the GO analysis, significant enrichment was observed in pathways related to photosynthetic electron transport in photosystem I (GO: 0009773), photosynthetic electron transport chain (GO: 0009767), photosynthesis, light harvesting in photosystem I (GO: 0009768), photosynthesis, light harvesting (GO: 0009765), and photosynthesis (GO: 0015979). These pathways were significantly regulated by ALA under NaCl stress (Figure 3C, Table S6). The KEGG enrichment analysis (Figure 3D) further confirmed that the common DEGs from the two comparisons were prominently expressed in key metabolic pathways, including photosynthesis-antenna proteins (map00196), photosynthesis (map00195), carbon fixation in photosynthetic organisms (map00710), and porphyrin metabolism (map00860). Collectively, the functional enrichment analysis indicated that ALA primarily regulated chlorophyll biosynthesis and photosynthesis in maize seedlings under salt stress.

### 2.4. Transcription Factor (TF) Family Analysis

**Figure 4:**
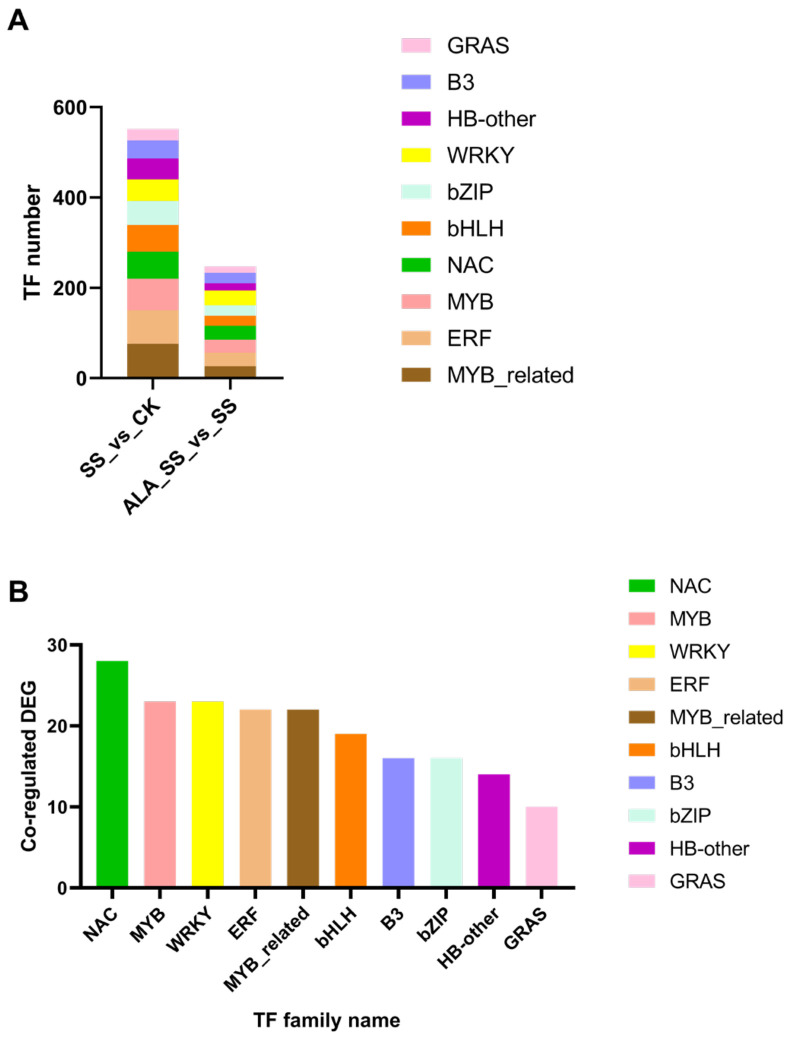
Analysis of transcription factors. (**A**) The stacked bar chart of the top 10 TF families in two comparisons. (**B**) The top 10 TF families’ number of co-regulated DEGs in two comparisons.

Transcription factors (TFs) play a critical role in the plant response to environmental stress. The DEGs from the SS_vs_CK comparison were associated with 45 TF families, with MYB-related, ERF, and MYB families being the most prevalent. In contrast, the DEGs from the ALA_SS_vs_SS comparison were linked to 37 TF families, with WRKY, NAC, and ERF families dominating. To further investigate these regulatory mechanisms, the study analyzed and identified the top 10 TF families shared between the two comparisons, including NAC, MYB, WRKY, MYB-related, ERF, bHLH, bZIP, B3, HB-other, and GRAS, all of which have been implicated in plant responses to stress (Figure 4, Table S7).

### 2.5. Porphyrin Metabolism and Photosynthesis-Antenna Proteins

**Figure 5:**
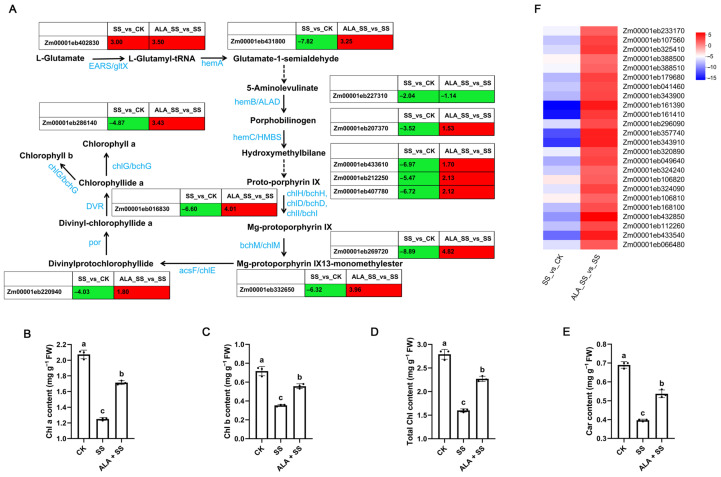
DEGs involved in porphyrin metabolism and photosynthesis-antenna proteins in two comparisons. The log_2_ fold change of FPKM represents the gene expression level in two comparisons. (**A**) Pathway of selected DEGs involved in porphyrin metabolism. The schematic pathways were modified according to KEGG enrichment. Solid arrows represent direct reactions. Dashed arrows represent indirect reactions. The red and green boxes express up- and down-regulated genes in two comparisons. (**B**) Statistics of Chl a content under different treatments. Different lowercase letters indicate a significant difference (*p* < 0.05). (**C**) Statistics of Chl b content under different treatments. (**D**) Statistics of total Chl content under different treatments. (**E**) Statistics of Car content under different treatments. (**F**) Heatmap of selected DEGs involved in photosynthesis-antenna proteins.

Chlorophyll, a key pigment in photosynthesis, is catalyzed by L-glutamate through a series of enzymes. As shown in Figure 5A and Table S8, one glutamyl-tRNA synthetase (EARS/gltX) DEG was up-regulated, while one porphobilinogen synthase (hemB/ALAD) DEG was down-regulated in both SS_vs_CK and ALA_SS_vs_SS comparisons. Several other DEGs encoding enzymes involved in chlorophyll biosynthesis, including glutamyl-tRNA reductase (hemA), hydroxymethylbilane synthase (hemC/HMBS), magnesium-chelatase (chlH/bchH, chlD/bchD, and chlI/bchI), magnesium-protoporphyrin O-methyltransferase (bchM/chlM), magnesium-protoporphyrin IX monomethyl ester (oxidative) cyclase (acsF/chlE), protochlorophyllide reductase (por), divinyl chlorophyllide a 8-vinyl-reductase (DVR), and chlorophyll synthase (chlG/bchG), were down-regulated in the SS_vs_CK comparison but up-regulated in the ALA_SS_vs_SS comparison. These results suggest that ALA treatment may enhance chlorophyll biosynthesis under salt stress.

Chlorophyll content in maize leaves decreased following NaCl stress (Figure 5B–E), with Chl a, Chl b, total Chl, and Car content decreasing by 39.61%, 51.39%, 42.65%, and 42.03%, respectively, compared to the untreated salt stress treatment, indicating that salt stress impaired the photosynthetic capacity of maize seedlings. However, compared to the NaCl treatment alone, ALA supplementation significantly increased Chl a, Chl b, total Chl, and Car content by 36.80%, 60.00%, 41.88%, and 35.00%, respectively, under salt stress.

Additionally, 24 light-harvesting chlorophyll proteins (LHC) were differentially expressed between the two comparisons. Seven light-harvesting complex I (LHCI) chlorophyll a/b binding protein DEGs (LHCA1, LHCA2, LHCA3, and LHCA4) and seventeen light-harvesting complex II (LHCII) chlorophyll a/b binding protein DEGs (LHCB1, LHCB2, LHCB3, LHCB4, LHCB5, and LHCB6) were significantly down-regulated in the SS_vs_CK comparison but were up-regulated in the ALA_SS_vs_SS comparison (Figure 5F, Table S9).

### 2.6. Photosynthesis

**Figure 6:**
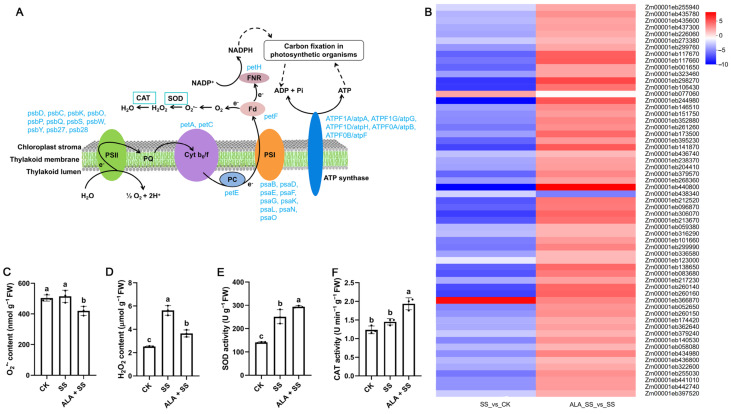
DEGs involved in photosynthesis in two comparisons. The log_2_ fold change of FPKM represents the gene expression level in two comparisons. (**A**) Pathway of selected DEGs involved in photosynthesis. The schematic pathways were modified according to KEGG enrichment. Solid arrows represent direct reactions. Dashed arrows represent indirect reactions. (**B**) Heatmap of selected DEGs involved in photosynthesis. (**C**) Statistics of O_2_^•–^ content under different treatments. Different lowercase letters indicate a significant difference (*p* < 0.05). (**D**) Statistics of H_2_O_2_ content under different treatments. (**E**) Statistics of SOD activity under different treatments. (**F**) Statistics of CAT activity under different treatments.

Photosynthesis is a key process in maize growth, and a total of 59 DEGs were enriched in the photosynthesis (map00195) across both comparisons (Figure 6A, Table S10). Among these, 22 DEGs involved in photosystem II (PSII) processes were identified, including two photosystem II P680 reaction center D2 proteins (psbD), one photosystem II CP43 chlorophyll apoprotein (psbC), one photosystem II PsbK protein (psbK), eleven photosystem II oxygen-evolving enhancer proteins (psbO, psbP, psbQ), one photosystem II 22kDa protein (psbS), two photosystem II PsbW proteins (psbW), two photosystem II PsbY proteins (psbY), one photosystem II Psb27 protein (psb27), and one photosystem II 13kDa protein (psb28). These DEGs were regulated in both comparisons. Furthermore, two apocytochrome f (petA) and two cytochrome b6-f complex iron–sulfur subunit (petC) DEGs were markedly down-regulated in SS_vs_CK but up-regulated in ALA_SS_vs_SS. Two plastocyanins (petE), which transfer electrons from the cytochrome b6/f complex, were also regulated in both comparisons. Additionally, photosystem I complex catalyzed electrons in the photosynthetic electron transfer chain; corresponding DEGs included psaB (2), psaD (1), psaE (2), psaF (2), psaG (2), psaK (1), psaL (2), psaN (1), and psaO (1), which were also regulated in both comparisons. Seven ferredoxin (petF) and four ferredoxin-NADP^+^ reductase (petH) genes showed varied expression patterns in the two comparisons, with some DEGs, such as Zm00001eb366870, being up-regulated across both comparisons, while others exhibited contrasting expression trends across the SS_vs_CK comparison. Lastly, seven ATP synthase genes, involved in ATP synthesis, were down-regulated in the SS_vs_CK comparison but showed up-regulation in the ALA_SS_vs_SS comparison. Gene expression levels are shown in the heatmap (Figure 6B).

### 2.7. Carbon Fixation in Photosynthetic Organisms

**Figure 7:**
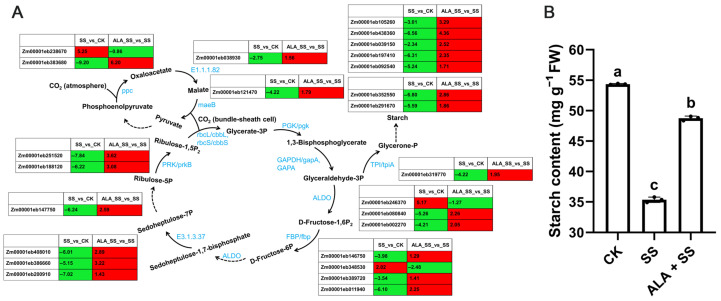
DEGs involved in carbon fixation in photosynthetic organisms in two comparisons. The log_2_ fold change of FPKM represents the gene expression level in two comparisons. (**A**) Pathway of selected DEGs involved in carbon fixation in photosynthetic organisms. The schematic pathways were modified according to KEGG enrichment. Solid arrows represent direct reactions. Dashed arrows represent indirect reactions. The red and green boxes express up- and down-regulated genes in two comparisons. (**B**) Statistics of starch content under different treatments. Different lowercase letters indicate a significant difference (*p* < 0.05).

The C_4_-dicarboxylic acid cycle is a fundamental pathway for carbon fixation in C_4_ plants. Significant changes in the expression of C_4_-cycle-related DEGs were observed across the two comparisons (Figure 7A, Table S11). Specifically, two phosphoenolpyruvate carboxylase (ppc) genes and two malate dehydrogenase (E1.1.1.82; maeB) genes were differentially expressed. Among the five ribulose-bisphosphate carboxylase genes, three ribulose-bisphosphate carboxylase large chain (rbcL/cbbL) and two ribulose-bisphosphate carboxylase small chain (rbcS/cbbS) genes were identified across the comparisons. The DEGs encoding phosphoglycerate kinase (PGK/pgk) were down-regulated in the SS_vs_CK comparison but up-regulated in the ALA_SS_vs_SS comparison. Furthermore, two distinct expression patterns of DEGs regulating glyceraldehyde 3-phosphate dehydrogenase were observed, including one GAPDH/gapA gene and two GAPA genes. Additionally, one triosephosphate isomerase (TPI/tpiA) gene, three fructose-bisphosphate aldolase (ALDO) genes, four fructose-1, 6-bisphosphatase I (FBP/fbp) genes, one sedoheptulose-bisphosphatase (E3.1.3.37) gene, and two phosphoribulokinase (PRK/prkB) genes, all key enzymes in carbon assimilation, were differentially expressed in the two comparisons. These genes contribute to the synthesis of sucrose or starch through the Calvin–Benson cycle.

In parallel, starch content in maize leaves under NaCl stress was significantly reduced by 34.96% compared to untreated plants, as shown in Figure 7B. However, exogenous ALA treatment under NaCl stress resulted in a substantial 37.87% increase in starch content, highlighting ALA’s potential to mitigate the negative impact of salt stress on starch accumulation.

### 2.8. O_2_^•−^ and H_2_O_2_ Levels and SOD and CAT Activities of Maize Response to Salt Stress by ALA

Oxygen molecules can be excited to a high-energy state by absorbing energy, thereby forming superoxide anions (O_2_^•–^) through photosynthesis. Salt stress induced the excessive production of ROS, including O_2_^•–^ and H_2_O_2_. O_2_^•–^ content increased by 2.20%, while H_2_O_2_ content rose significantly by 123.02% compared to untreated plants. However, the ALA + SS treatment significantly reduced O_2_^•–^ and H_2_O_2_ levels by 18.49% and 35.05%, respectively, compared to the salt stress alone (Figure 6C,D). Additionally, SOD and CAT activities in maize leaves were markedly higher in the CK treatment, showing increases of 78.42% and 17.89%, respectively, compared to the salt stress treatment. These enzyme activities were further enhanced by 16.97% and 33.10%, respectively, under exogenous ALA treatment with NaCl stress compared to the salt stress treatment alone (Figure 6E,F).

### 2.9. qRT PCR Verification of RNA-Seq Data

**Figure 8:**
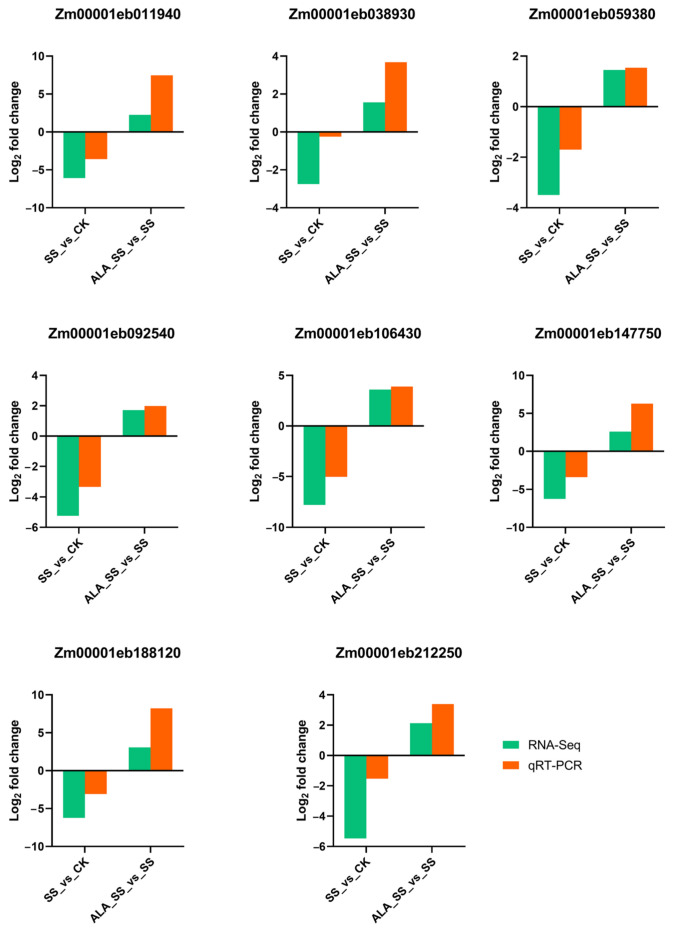
Comparisons of log_2_ fold change of 8 selected DEGs in RNA-Seq and qRT-PCR results.

To validate the RNA-Seq results, the expression levels of eight DEGs, including Zm00001eb011940 (FBP/fbp), Zm00001eb038930 (E1.1.1.82), Zm00001eb059380 (psaF), Zm00001eb092540 (rbcS/cbbS), Zm00001eb106430 (psbQ), Zm00001eb147750 (E3.1.3.37), Zm00001eb188120 (PRK/prkB), and Zm00001eb212250 (chlD/bchD), which were enriched in photosynthetic-related pathways, were further analyzed using qRT-PCR. The qRT-PCR data confirmed that the expression patterns of the selected DEGs between the two comparisons closely mirrored those obtained from RNA-Seq (Figure 8), reinforcing the reliability of the transcriptome data.

## 3. Discussion

The sensitivity of maize to salt stress has become a major limiting factor for its growth and development [1,6], resulting in significant suppression of leaf growth and plant height [26]. Application of ALA has been shown to significantly enhance plant growth [27]. In this study, salt stress treatment led to plant dwarfing and leaf shrinkage in maize seedlings, while exogenous ALA alleviated these adverse effects (Figure 1A). To investigate the role of ALA in modulating the salt tolerance mechanisms of maize, transcriptome analyses were performed on maize seedlings under three conditions: CK, SS, and ALA_SS. The results revealed 4634 co-expressed DEGs between the two comparisons (SS_vs_CK and ALA_SS_vs_SS), forming the foundation for exploring the molecular mechanisms through which ALA regulates salt tolerance in maize (Figure 2E). GO analysis indicated that the majority of DEGs were involved in photosynthesis, light harvesting, and photosynthetic electron transport (Figure 3C). KEGG enrichment analysis identified key pathways related to ALA’s regulation of salt stress in maize, including photosynthesis, carbon fixation in photosynthetic organisms, and porphyrin metabolism (Figure 3D). ALA plays a pivotal role in the biosynthesis of chlorophyll and cytochrome in plants, which subsequently increases the net photosynthetic rate of leaves, enhancing light-harvesting efficiency, strengthening photosynthesis, and improving plant stress resistance [17,28]. Therefore, this study focused on the impact of ALA on salt stress in maize from the perspective of photosynthesis.

ALA serves as a precursor in the biosynthesis of chlorophyll, synthesized from glutamate through the sequential catalysis by glutamyl tRNA synthetase, glutamyl tRNA reductase, and glutamate-1-semialdehyde aminotransferase. ALA is an essential compound for enhancing chlorophyll biosynthesis in plants [29]. The critical step in chlorophyll synthesis involves the insertion of Mg^2+^ into proto-porphyrin IX by magnesium chelatase, forming Mg-protoporphyrin IX [30]. Additionally, protochlorophyllide, formed during chlorophyll biosynthesis, is subsequently catalyzed by protochlorophyllide reductase and chlorophyll synthase to yield chlorophyllide and chlorophyll a [31]. Exogenous ALA has been shown to regulate the transcription levels of biosynthesis-related genes such as *HEMA1*, *HEMB*, and *FAR1* in pepper seedlings under cold stress, thereby enhancing the chlorophyll biosynthesis pathway [18]. In this study, 18 DEGs enriched in the porphyrin metabolism pathway were identified across two comparisons, including hemA, hemB, EARS, por, chlG, and other key genes (Figure 5A and Table S8), all of which are involved in chlorophyll biosynthesis. Salt-stress-induced damage to the chloroplast ultrastructure impairs stomatal conductance and photosynthetic carbon assimilation [32], while exogenous ALA preserves the stability of chloroplast ultrastructure [33]. The microstructure of maize seedling leaves was found to be damaged under salt stress, whereas ALA supplementation maintained cellular integrity (Figure 1B), which likely supports more effective photosynthesis. Salt stress was shown to reduce the contents of Chl and Car [34], and it had been reported that a reduction in endogenous ALA synthesis significantly diminishes chlorophyll content in NaCl-stressed plants [32]. Therefore, exogenous ALA application proved effective in enhancing chlorophyll content [34]. Consistent with these observations, ALA treatment increased Chl and Car contents in maize leaves under salt stress (Figure 5B–E).

Light-harvesting chlorophyll a/b binding (LHC) proteins (LHCA and LHCB), which are closely associated with photosystem (PS) I and II, play a pivotal role in chlorophyll degradation and in maintaining the greenness of plants [35]. The LHC gene encodes proteins that bind to pigment molecules to form light-harvesting chlorophyll protein complexes, crucial for light energy absorption and transport [36]. The LHC gene family in wheat has been implicated in salt tolerance [37]. In this study, 24 light-harvesting chlorophyll a/b binding protein DEGs were identified, which were down-regulated in salt-treated plants but up-regulated in ALA-treated maize seedlings under NaCl stress (Figure 5F and Table S9), suggesting that ALA regulates photosynthesis and enhances salt tolerance in maize.

PSII is a key complex involved in photosynthetic electron transfer, utilizing energy from light to split water into protons and electrons while releasing oxygen. The oxygen-evolving complexes in its reaction center serve as the active sites for photosynthetic water oxidation [38]. Under salt stress, ALA modulated 22 PSII assembly factors (Figure 6A,B and Table S10), indicating that transcriptional regulation of related genes contributes to the repair of damaged PSII [39]. Maintaining cytochrome b6/f complex activity ensures the balance of electron transfer between PSI and PSII [40]. In this study, up-regulation of cytochrome complex genes petA and petC in ALA_SS_vs_SS facilitated adjustments in the electron transport chain, promoting photoprotective effects [40]. Additionally, two plastocyanin (petE), seven ferredoxin (petF), and four ferredoxin-NADP^+^ reductase (petH) genes were expressed across both comparisons, acting as primary carriers in photosynthetic electron transfer. These genes help transfer electrons to NADP^+^, reducing it to NADPH, which, in conjunction with ATP, is utilized in the Calvin–Benson cycle for CO_2_ fixation into organic compounds [41]. ATP is generated by ATP synthase through the conversion of ADP and Pi, with regulation observed across comparisons.

Disruptions to electron flux balance in the electron transport chain under light stress can lead to the excessive accumulation of ROS, causing photo-oxidative damage [40]. Salt stress resulted in the production and over-accumulation of ROS (O_2_^•–^ and H_2_O_2_) in maize seedling cells (Figure 6C,D), triggering oxidative stress. SOD and CAT, as primary antioxidant defense enzymes, suppress ROS generation by converting O_2_^•–^ to H_2_O_2_, which is then decomposed into O_2_ and H_2_O [42]. SOD and CAT activities were found to increase in maize seedling leaves under salt stress (Figure 6E,F), suggesting a compensatory response to mitigate ROS levels and adapt to environmental stress. Under salt stress, exogenous ALA reduced ROS content and enhanced antioxidant enzyme activities, advancing ROS clearance and preserving cell membrane integrity [21].

Phosphoenolpyruvate carboxylase, a pivotal carboxylase involved in CO_2_ fixation, is essential for the C_4_ pathway and is expressed during leaf greening [43], playing a critical role in environmental stress tolerance [44]. In this study, two phosphoenolpyruvate carboxylase genes and two malate dehydrogenase genes were differentially regulated in the two comparisons (Figure 7A and Table S11). Malate dehydrogenase is involved in various biochemical and physiological processes in plants, including the C_4_ cycle. Previous research has shown that malate dehydrogenase gene expression supports plant and cell growth, as well as responses to salt stress [45]. Photosynthesis, the process by which plants use solar energy to convert CO_2_ into organic compounds via enzyme-catalyzed reactions in the Calvin–Benson cycle, is fundamental for plant growth [46]. In this study, key catalytic enzyme genes, such as those for ribulose-bisphosphate carboxylase, phosphoglycerate kinase, glyceraldehyde 3-phosphate dehydrogenase, triosephosphate isomerase, fructose-bisphosphate aldolase, fructose-1, 6-bisphosphatase I, sedoheptulose-bisphosphatase, and phosphoribulokinase, were regulated differently across the two comparisons. Glyceraldehyde 3-phosphate, a product of the photosynthetic carbon cycle, can be retained in the chloroplasts for starch synthesis [41]. High salt stress impairs starch synthesis by reducing water potential, inhibiting photosynthesis, and altering the expression of starch synthesis genes [47,48]. Salt stress significantly reduced starch content, whereas ALA supplementation enhanced starch accumulation (Figure 7B). ALA may help sustain the metabolic balance of carbohydrate products, thereby improving stress resistance [27].

Functional genes are regulated by specific TFs, which activate self-protection mechanisms to help plants cope with salt stress [49]. NAC TFs, for instance, enhance tolerance to abiotic stresses by activating stress-responsive genes. Overexpressing *OsNAC23* plants exhibit higher photosynthetic rates and sugar transport, thereby increasing rice yield [50]. MYB TFs are widely distributed in higher plants and play essential roles in plant development and responses to abiotic stresses [51]. Studies have demonstrated that overexpression of MdMYB108L enhances the plant’s photosynthetic ability under salt stress [52]. WRKY TFs, one of the largest TF families in plants, play a critical regulatory role in sugar metabolism and photosynthesis processes [53]. MYB-related TFs have been shown to play a significant role in mediating plant responses to abiotic stress, with overexpression of *AhMYB30* enhancing the ability to maintain green leaves, which may enhance photosynthesis and improve salt tolerance in transgenic plants [54]. TF families, including NAC, MYB, WRKY, and bZIP, have been identified as being linked to abiotic stress responses, and some TF genes can regulate genes involved in the photosynthetic light reaction pathway to improve plant photosynthetic capacity [55]. In the current study, the top 10 TF families identified in the two comparisons were NAC, MYB, WRKY, MYB-related, ERF, bHLH, bZIP, B3, HB-other, and GRAS (Figure 4, Table S7). These observations provide a foundation for understanding the photosynthetic molecular mechanisms underlying salt tolerance in maize.

## 4. Materials and Methods

### 4.1. Plant Materials and Stress Treatments

Maize (*Zea mays* L.) seeds of the “Zhengdan 958” cultivar, sourced from the Henan Academy of Agricultural Sciences, were used for the salt stress experiments at Suzhou University. Uniform seeds were disinfected with 2.5% NaClO solution for 10 min, followed by rinsing with distilled water three times. Imbibed seeds were then germinated in a germination box (19 × 13 × 12 cm) lined with wet filter paper and placed in an artificial illumination incubator for 7 days at a day/night temperature of 28/20 °C, a 14/10 h light/dark cycle, with a light intensity of 400 μmol m^−2^ s^−1^ and relative humidity maintained at 65–70%. Four seedlings with consistent growth were fixed with perforated pearl cotton foam and placed in individual culture bottles (10.8 cm high, 7.5 cm diameter, and 6.9 cm caliber) containing 1/2 Hoagland solution (pH 6.5). At the three-leaf stage, maize seedlings were sprayed with 40 mg/L, 5-aminolevulinic acid (ALA) or distilled water (control). Approximately 48 h post-spraying, two-thirds of the seedlings were subjected to salt stress, as detailed below: (1) 0 mg/L ALA + 0 mmol/L NaCl, (2) 0 mg/L ALA + 300 mmol/L NaCl, and (3) 40 mg/L ALA + 300 mmol/L NaCl. The second leaf of maize plants was sampled 72 h after salt stress treatment. Physiological indexes were extracted from three biological replicates of fresh samples (three treatments were labeled as CK, SS, and ALA + SS, respectively), while transcriptome analysis was conducted on three biological replicates of frozen samples (three treatments were labeled as CK, SS, and ALA_SS, respectively) stored at −80 °C.

### 4.2. Chlorophyll Contents and Starch Content Determination

Chl content (Chl a, Chl b, total Chl, and carotenoid [Car]) in maize leaves was measured using a slightly modified procedure from Arnon [56]. A 0.1 g fresh leaf sample was cut and immersed in 10 mL of anhydrous ethanol until the leaves became colorless under darkness. The absorbance of the filtrate was measured at 663, 645, and 470 nm using ethanol as a reference for zero adjustment. The Chl contents were calculated using the following formulas: Chl a (C_a_) = 12.7D_663_ − 2.69D_645_; Chl b (C_b_) = 22.9D_645_ − 4.68D_663_; total Chl = 20.2D_645_ + 8.02D_663_; Car = (1000D_470_ − 3.27C_a_ − 104C_b_)/229.

Starch content was measured following the protocols of the Starch Content Reagent Kit (Cat#BC0700) from Beijing Solarbio Science & Technology Co., Ltd., Beijing, China.

### 4.3. Observation of Microstructure in Leaves

Histological analysis was performed using paraffin sections. Functional leaves from different treatments were selected, and 5 mm × 5 mm samples near the 1/3 position of the main vein were immediately placed into centrifuge tubes containing FAA (formalin–acetic acid–alcohol mixture) fixative. Fixed leaves were dehydrated sequentially with different concentrations of alcohol, and then organizational transparency was conducted with transparent agents. The transparent tissue blocks were sequentially impregnated with 3 cylinders of paraffin wax (60 °C) and wrapped in paraffin blocks. Paraffin-embedded tissue blocks were sliced and attached. Finally, the paraffin sections were dewaxed, stained with toluidine blue dye, and sealed. The stained slices were observed under a biological microscope (Olympus Corporation, Tokyo, Japan).

### 4.4. Reactive Oxygen Metabolism

The hydrogen peroxide (H_2_O_2_) content in maize leaves was measured using the potassium iodide method [57]. The superoxide anion content was determined based on the hydroxylamine oxidation method as described by Elstner and Heupel [58]. The activity of superoxide dismutase (SOD) in maize leaves was estimated by the nitro-blue tetrazolium (NBT) photochemical reduction method [59] with minor modifications. Catalase (CAT) activity was assessed using an improved UV absorption method based on the protocol proposed by Beers and Sizer [60].

### 4.5. Total RNA Isolation, Library Construction, and Transcriptome Sequencing

Total RNA from maize seedling leaves was separated and purified with TRIzol^®^ Reagent on the basis of the manufacturer’s protocol. The concentration and purity of total RNA was detected by Nanodrop2000 (NanoDrop, Wilmington, DE, USA). Agarose gel electrophoresis was used to check the integrity of RNA, and Agilent5300 (Agilent, CA, USA) was used for determining the RNA quality number (RQN) value. The library construction required RNA with a total amount of 1 μg, concentration ≥ 30 ng/μL, RQN > 6.5, and OD260/280 between 1.8 and 2.2.

mRNA was isolated from total RNA using magnetic beads with Oligo (dT) for A–T base pairing, followed by random fragmentation into approximately 300 bp. cDNA was synthesized through reverse transcription, and the final library was obtained through PCR amplification. mRNA sequencing was based on second-generation high-throughput sequencing technology and carried out on the NovaSeq X Plus platform (Majorbio Bio-pharm Biotechnology Co., Ltd., Shanghai, China). The raw sequencing data have been submitted to the NCBI Sequence Read Archive (SRA) database under the registered number PRJNA1126090.

### 4.6. RNA-Seq Analysis and Identification of Differentially Expressed Genes

Quality control of the raw data was performed using fastp software (v0.19.5), which filtered out joint sequences, low-quality sequences, and reads containing ’N’. The purified high-quality reads were mapped to the *Zea mays* L. genome (Zm-B73-REFERENCE-NAM-5.0) using HISAT2 [61]. Gene expression levels were quantified using RSEM software (Version 1.3.3) [62] across different samples. DEGs were identified using DESeq2 software (Version 1.24.0) [63] to explore their functional roles. Genes with a fold change ≥ 2 and a false discovery rate (FDR) < 0.05 were considered significantly differentially expressed. Gene Ontology (GO) and Kyoto Encyclopedia of Genes and Genomes (KEGG) functional enrichment analyses were performed using Goatools (Version 0.6.5) and Python SciPy (Version 0.13.3) software, respectively. DEGs significantly enriched in GO terms and metabolic pathways were identified with a corrected *p*-value < 0.05.

### 4.7. Quantitative Real-Time PCR Identification

Reverse transcription of the extracted and purified RNA was performed using the FastKing RT Kit (Tiangen BioTek, Beijing, China) to synthesize cDNA for quantitative real-time PCR (qRT-PCR). The qRT-PCR was conducted with Power qPCR PreMix (Genecopoeia, Rockville, MD, USA) in a 20 μL reaction volume containing 10 µL of SYBR Green Mix, 0.5 µL of each primer (forward and reverse), 1 µL of cDNA, and 8 µL of H_2_O, following this reaction protocol: 95 °C for 600 s, then 40 cycles of 95 °C for 10 s and 60 °C for 40 s. Forward and reverse primers were designed using Primer Premier (Version 6), as detailed in Supplementary Table S12. GAPDH was used as the internal reference gene. The relative expression levels of each target gene were calculated using the 2^−ΔΔCt^ method [64].

### 4.8. Statistical Analysis

IBM SPSS Statistics 20 software was employed for statistical analysis of the data between samples, using one-way analysis of variance (ANOVA) followed by Duncan’s multiple range test, with a significance threshold set at *p* < 0.05. Graphics and heatmaps were generated using GraphPad Prism 8.0 software and the Majorbio Cloud Platform, respectively.

## 5. Conclusions

This study provides a comprehensive analysis of the transcriptomic changes in maize seedlings under salt stress with exogenous ALA application, contributing to the understanding of the molecular mechanisms by which ALA enhances salt tolerance in maize. In summary, ALA improves photosynthesis, mitigating and delaying the damage caused by salt stress to maize seedlings. Morphological data indicated that ALA alleviated the detrimental effects of salt stress on maize seedling growth and preserved the integrity of leaf mesophyll cellular structure. RNA-Seq analysis revealed that 4634 DEGs were co-expressed between the two comparisons (SS_vs_CK and ALA_SS_vs_SS). KEGG enrichment analysis showed that these DEGs were primarily enriched in pathways related to porphyrin metabolism, photosynthesis-antenna proteins, photosynthesis, and carbon fixation in photosynthetic organisms. Physiological assays indicated the following: 1) ALA inhibited the degradation of photosynthetic pigments under salt stress, which facilitated the continuation of effective photosynthesis; 2) ALA enhanced the activities of SOD and CAT, aiding in the removal of excessive ROS (O_2_^•–^ and H_2_O_2_) accumulated under salt stress; and 3) ALA mitigated the decrease in starch content under salt stress. TF family analysis identified NAC, MYB, WRKY, and MYB-related TFs as key regulators in the two comparisons. These findings offer valuable insights into the alleviation mechanisms of ALA on salt stress in maize seedlings, particularly from the perspective of photosynthesis, which may have significant impacts on the cultivation of maize, including growth and development, resistance, as well as yield and quality.

## Data Availability

The RNA-Seq data presented in the study are deposited in the NCBI repository under accession number PRJNA1126090. The referenced data in this study are included in the article.

## Supplementary Materials

The following supporting information can be downloaded at: https://www.mdpi.com/article/10.3390/ijms26020786/s1.
