# Supplementary material for: Analysis of the Transcriptome Provides Insights into the Photosynthate of Maize Response to Salt Stress by 5-Aminolevulinic Acid
Source: Int J Mol Sci. 2025 Jan 17;26(2):786. doi: 10.3390/ijms26020786 (PMC11765576; doi:10.3390/ijms26020786)
Supplement: Supplementary file 1 [file ijms-26-00786-s001.zip › Supplementary Figure S1.pdf]

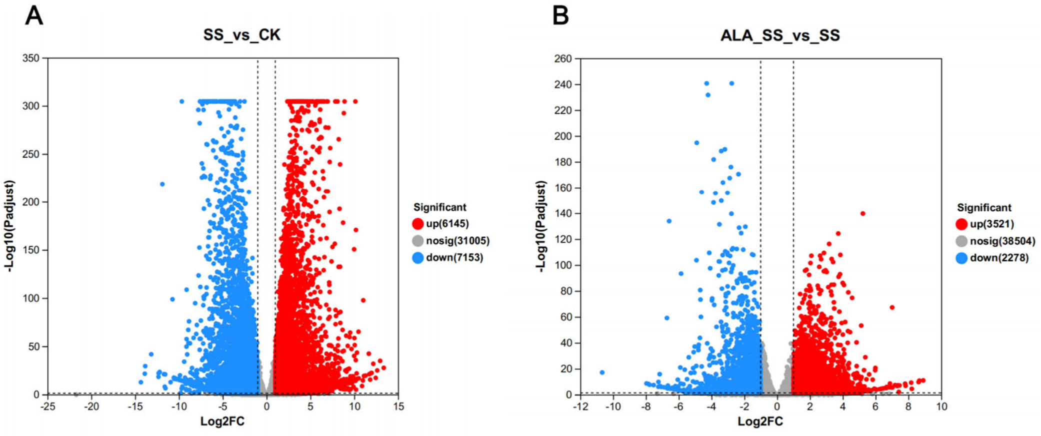

Figure S1. Volcano diagram of DEGs in diverse comparisons. (**A**) Volcano plot of SS_vs_CK; (**B**) Volcano plot of ALA_SS_vs_SS;
